# Right Tail of the Distribution of Depressive Symptoms Is Stable and Follows an Exponential Curve during Middle Adulthood

**DOI:** 10.1371/journal.pone.0114624

**Published:** 2015-01-14

**Authors:** Shinichiro Tomitaka, Yohei Kawasaki, Toshiaki Furukawa

**Affiliations:** 1 Department of Mental Health, Panasonic Health Center, Tokyo, Japan; 2 Department of Mathematics, Tokyo University of Science, Tokyo, Japan; 3 Department of Health Promotion and Human Behavior, Kyoto University Graduate School of Medicine/School of Public Health, Kyoto, Japan; Institute of Psychiatry, UNITED KINGDOM

## Abstract

**Background:**

Previous research has reported that the mean of depressive symptoms is stable in the general population through middle adulthood. To understand the stability of depressive symptoms during middle adulthood, we investigated the nature of the distribution of depressive symptoms.

**Methods:**

We analyzed 24,890 subjects aged 15 to 84 years who participated in the Active Survey of Health and Welfare, Japan. Depressive symptoms were assessed using the Japanese version of the Center for Epidemiologic Studies Depression Scale (CES-D). The descriptive statistics and frequency curves of the distributions were then compared according to age group.

**Results:**

The distribution of depressive symptoms was stable through middle adulthood. The right tail which covers clinical depression was more stable than the left tail or peak of the distributions. The right tail of the distribution during middle adulthood exhibited a linear pattern with a log-normal scale.

**Conclusions:**

The right tail of the distribution of depressive symptoms is stable and exhibits an exponential pattern during middle adulthood.

## Introduction

Depression is a common mental disorder that is among the leading causes of disability worldwide [1]. Given that depressive symptoms are closely linked with depression, there has been a great interest in understanding differences in depressive symptoms according to patient age.

Although there are a few exceptions, cross-sectional surveys and longitudinal studies have found that the trajectory of depressive symptoms across the adult lifespan follows a U-shaped pattern, with symptoms being high during young adulthood, decreasing during middle adulthood, and then increasing again during older age [2, 3, 4, 5]. A number of explanations have been proposed for this finding, with most focusing on possible factors explaining the increases in depressive symptoms during young adulthood and older age [6].

While depressive symptoms follow a U-shaped pattern across adulthood, age-related changes in depressive symptoms seem relatively mild. The estimated average change per decade in the Center for Epidemiological Studies Depression Scales (CES-D) score is less than 1 point between the ages of 20 and 70 years, whereas the standard deviation of CES-D scores in community surveys is between 5 to 10 points [3, 7]. Thus, age-related changes in the mean CES-D score during adulthood are relatively small in comparison with individual differences. Between the ages of 30 and 60 years, in particular, the mean CES-D scores appear to stabilize [3, 8]. Taking the individual variability of depressive symptoms and vulnerability to depression into account, the stability of the mean CES-D scores during middle adulthood should be emphasized.

While a lot of attention has been paid to the increase in depressive symptoms during young adulthood and older age, little attention has been given to the stability of depressive symptoms during middle adulthood. However, an investigation of the stability of depressive symptoms is similarly important to understand the course of depression. The stability of the mean of depressive symptoms indicates that depressive symptoms recover on average. Furthermore, the stability of depressive symptoms suggests that depressive symptoms are steadily regulated in some way. A certain rule may exist in the distribution of depressive symptoms during middle adulthood. To understand the stability of depressive symptoms, the nature of the distribution of depressive symptoms must be characterized.

The present study used the depressive symptoms assessment of the Active Survey of Health and Welfare, which has been conducted annually to evaluate the health status of a representative sample of the Japanese general population [9]. Using more than 20,000 CES-D assessments performed in subjects between the ages of 15 and 84 years, we examined the similarity of the distribution of depressive symptoms according to different ages. The descriptive statistics and frequency curves of the distributions were then compared according to age group. We then delineated the pattern of the right tail of the distribution of depressive symptoms.

## Methods

We used data from the Active Survey of Health and Welfare (ASHW) conducted by the Japanese Ministry of Health, Labor and Welfare in 2000 [9]. The ASHW is an annual nationwide survey conducted to obtain data required for policy making by the Japanese Government. In 2000, the ASHW examined depressive symptoms among a representative sample of the Japanese general population. To ensure that the sample was representative of the general population, survey participants were selected from among individuals aged >12 years living in 300 communities in Japan. These communities were selected from 881,851 precincts identified in the 1995 Census using a stratified sampling design. This study was approved by the ethics committee of Panasonic Health Center. Oral informed consent was obtained from all the subjects. The data and methods used by the survey were described in detail [9].

The questionnaire was returned by 32,729 respondents. The response rate was not publicized by the Ministry of Health, Labor and Welfare and Health. However, the response rates for similar surveys conducted 3 and 4 years earlier were 87.1% and 89.6% [10]. We assumed that the response rate for the present study was over 80%. We excluded 6,589 respondents with missing information on one or more key study variables (i.e., depressive symptoms or age). In order to ensure reliability of self-reported assessments, we further excluded 1,248 respondents who were younger than 15 years or older than 85 years. The final sample consisted of 24,890 respondents between the ages of 15–84 years.

### Measures

Depressive symptoms were assessed using the Japanese version of the Center for Epidemiologic Studies Depression Scale (CES-D). This 20-item scale assesses the frequency of a variety of depressive symptoms within the previous week (0 = rarely or none of the time, 1 = some of the time, 2 = much of the time, and 3 = most or all of the time) [11]. High values on the scale indicate greater psychological distress. Previous surveys have consistently reported that the mean CES-D scores in East Asia are higher than those of Western countries [12]. While a score of 16 is typically considered to be the threshold for clinical depression in Western countries, a score of 25 is frequently defined as the threshold for depression in Eastern Asia [13].

### Analysis procedure

The respondents were grouped into the following age groups: 15–19 years, 20–29 years, 30–39 years, 40–49 years, 50–59 years, 60–69 years, 70–79 years, and 80–84 years. Descriptive statistics, such as the mean, standard deviation, skewness, kurtosis, and frequency curve, were calculated for each age group. To estimate the proportion of high CES-D scores, we calculated the 90th percentile of the CES-D score for each group.

The first step in this analysis was to ascertain the U-shaped effect by comparing the mean CES-D of each age group. After confirming the U-shaped-effect, we compared the frequency curve among a young adult group (15–19 years, 20–29 years, 30–39 years), a middle adulthood group (30–39 years, 40–49 years, 50–59 years, 60–69 years), and an older age group (60–69 years, 70–79 years, and 80–84 years). After confirming that the middle adulthood group to be the most stable among the three groups and the right tails of the distributions which cover clinical depression were more stable than the left tails or peaks of the distributions, the right tails of the distributions for middle adulthood were analyzed using a log-normal scale to examine the similarities over a wide range of CES-D scores. Finally, to illustrate the patterns of the frequency of depressive symptoms during middle adulthood, the tail of the distribution, including the ages between 30–69 years, was evaluated using a log-normal scale. We used JMP version 11 for Windows to calculate the descriptive statistics and the frequency distribution curve.

## Results

**Figure 1:**
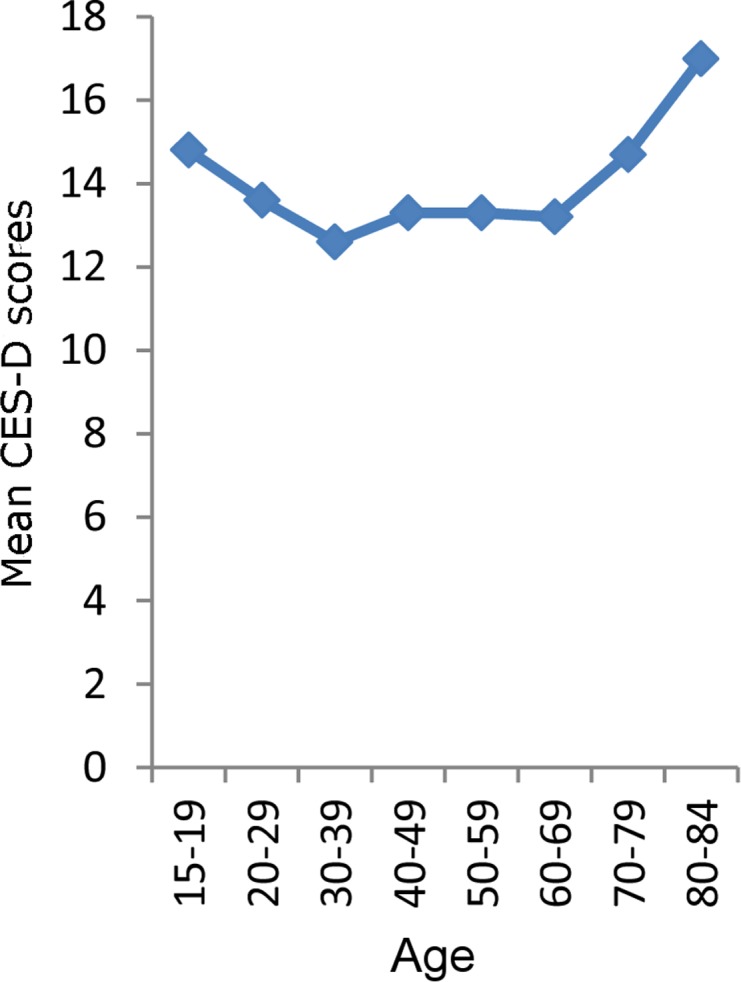
Relationship between age and the mean CES-D score.

**Figure 2:**
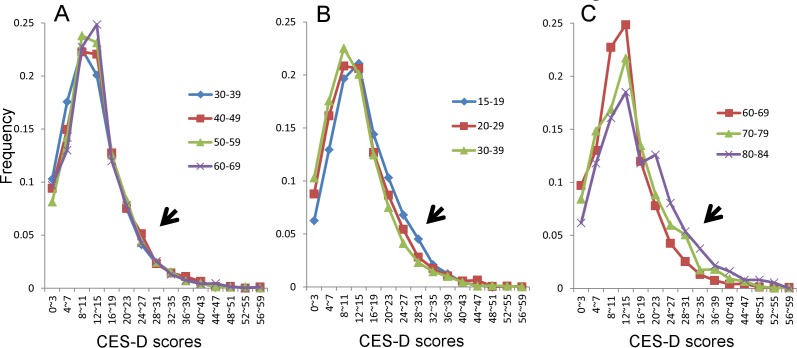
CES-D distributions for the middle adulthood group (A), the young adulthood group (B), and the older age group (C).

The descriptive statistics of the distributions of CES-D in the Japanese general population according to age groups are shown in Table 1. As depicted in Fig. 1, the distributions of depressive symptoms exhibited a U-shaped pattern, with depressive symptoms being high during adolescence and young adulthood, maintaining stability during middle adulthood, and then increasing during older ages, which is consistent with previous reports [3]. Next, the distributions of the CES-D scores were compared among the young adult group (15–19 years, 20–29 years, and 30–39 years), the middle age group (30–39 years, 40–49 years, 50–59 years, and 60–69 years), and the older age group (60–69 years, 70–79 years, and 80–84 years). Fig. 2 shows that the distribution of CES-D in the middle adulthood group was the most stable. As indicated by the arrows, the right tails of the curve were similar to each other among middle adulthood group (Fig. 2A), whereas the right tails of the young adult group (Fig. 2B) and the old age group (Fig. 2C) differed from each other. When the similarity of the frequency curve during middle adulthood was compared according to the components of the distribution, such as the peak, right tail, and left tail, the right tails were more stable than the left tails or peaks of the distributions. In support of the stability of the right tails, the 90th percentile of the CES-D scores was almost the same for the 30–39 year and 60–69 year age groups.

**Table 1:** Summary of descriptive statistics for the CED-D distribution in the general Japanese population according to age group.

**Age group**	**Number**	**Female (%)**	**Mean ± S.D.**	**Skewness**	**kurtosis**	**Medium**	**90^th^ percentile**
15–19	1854	48	14.8 ± 8.4	0.76	0.69	13	27
20–29	4275	53	13.6 ± 8.6	1.04	1.46	12	25
30–39	4334	53	12.6 ± 8.1	1.10	1.91	11	23
40–49	4408	52	13.3 ± 8.3	1.19	2.46	12	24
50–59	4785	52	13.3 ± 7.9	1.09	2.10	12	23
60–69	3198	51	13.2 ± 8.1	1.18	2.63	12	23
70–79	1663	56	14.7 ± 9.3	0.97	1.05	13	28
80–84	373	75	17.0 ±10.4	0.88	0.80	15	31

**Figure 3:**
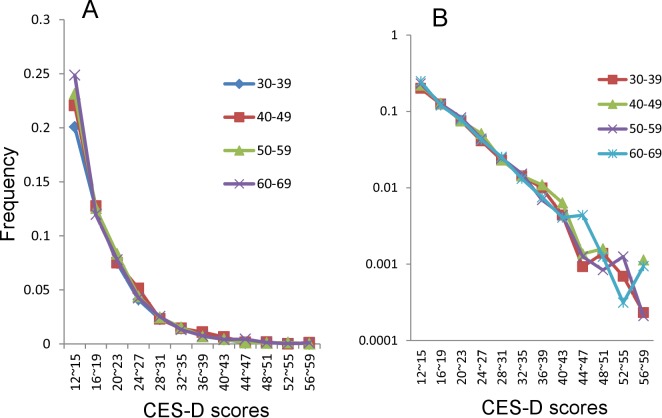
The right tails of the distributions for middle adulthood were compared using a normal scale (A) and a log-normal scale (B).

As shown in Fig. 3A, the right tails of the distributions for middle adulthood were similar to a normal scale. To examine the similarities over a wide range of CES-D scores, the right tails of the distributions for middle adulthood were compared using a log-normal scale (Fig. 3B). In the range of CES-D scores from 12–15 points to 40–43 points, the curves for each age group exhibited a linear pattern. However, for CES-D scored over 40–43 points, the curves for each age group fluctuated randomly, reflecting small sample sizes in each age group.

**Figure 4:**
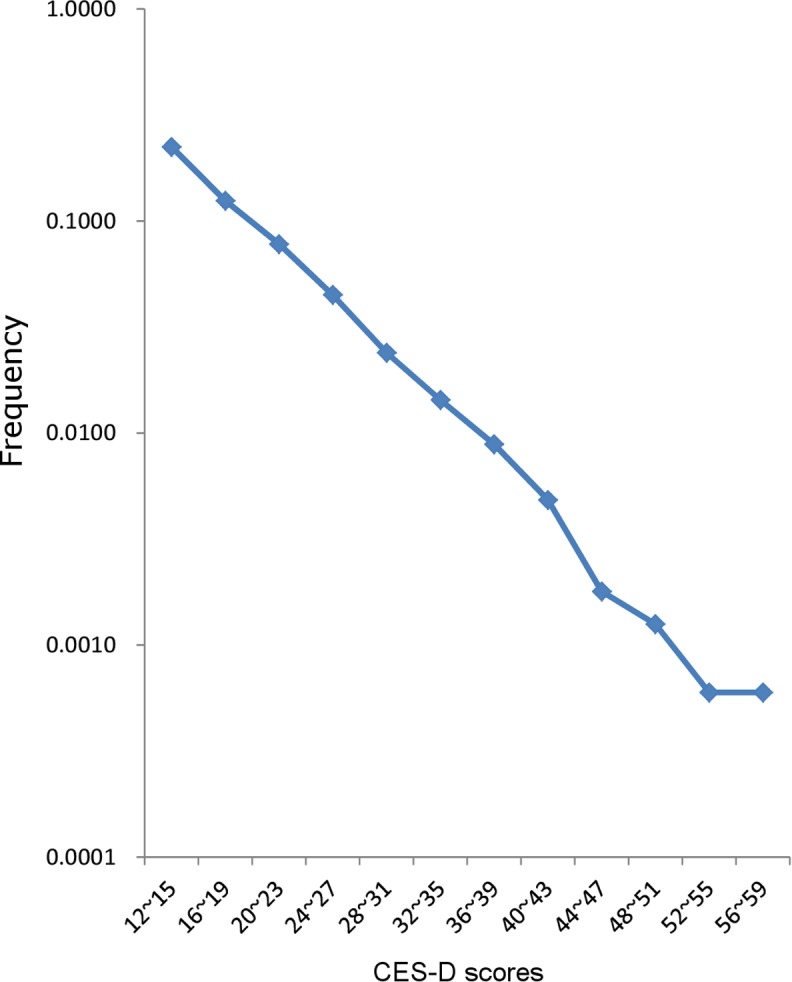
The right tail of the distributions, including all respondents between the ages of 30–69 years, plotted using a log-normal scale.

To demonstrate the patterns of the distribution of depressive symptoms during middle adulthood, the right tail of the distribution, including all ages from 30 to 69 years (N = 16,725), was evaluated using a log-normal scale (Fig. 4). The right tail of the distribution for middle adulthood showed a linear pattern with a log-normal scale.

## Discussion

The aim of the present study was to investigate the stability of depressive symptoms during middle adulthood by analyzing the distributions of CES-D scores in the general population according to different age groups. Our findings showed that the distribution of depressive symptoms, especially the right tail of the distribution, was stable among middle adulthood group. A number of studies have found that the mean of depressive symptoms stabilizes during middle adulthood [3, 4]. Our findings indicate that the stability of depressive symptoms for middle adulthood group is based on the stability of the right tail of the distribution. The stability of the right tail is noteworthy because the right tail covers clinical depression.

Interestingly, during middle adulthood, when the distribution of depressive symptoms is most stable, the right tail of the distribution of the CES-D scores exhibits a linear pattern with a log-normal scale, suggesting that the right tail of the distribution of depressive symptoms follows an exponential curve. In accordance with our results, Meltzer et al. (2002) reported that an exponential curve provided the best fit for total neurotic symptoms and depressive scores from British National Survey of Psychiatric Morbidity, but floor effects produced deviations at symptom counts of 0–3 [14]. After truncation, an exponential curve fitted the symptoms data excellently.

The reason why the right tail of the distribution of depressive symptoms follows an exponential curve is unknown, but the conditions that enable such a distribution can be speculated upon. Exponential distribution is observed where individual variability and total stability are organized together, such as the barometric formula and the Boltzmann-Gibbs law [15]. In more detail, the atmospheric pressure (or density) decreases exponentially with an increasing height above sea level, a relation that is known as the barometric formula. The barometric formula implies that atmosphere is distributed in order, while circulating dynamically.

To form the exponential distribution of atmosphere pressure with altitude, three factors are needed: the random movement of gas molecules, the force of gravity, and a boundary (the surface of the ground). In other words, an exponential distribution is formed when these three conditions are satisfied. In the case of depressive symptoms, if individual depressive symptoms corresponding to the random movements that comprise the individual variability could be demonstrated and both a boundary and the force to a boundary that constitute the stability of the distribution could be identified, the reason why depressive symptoms follow an exponential curve could possibly be explained. Further consideration regarding this speculation is needed.

This study had some limitations. First, people with depression might have been less likely than others to participate in the survey. Evidence suggests that a bias toward a reluctance to participate exists for psychiatric epidemiological surveys, although our findings are consistent with cross-sectional studies of age-related associations in depressive symptoms [16]. Second, this study did not examine any potential mechanisms, or mediators, underling the stability of the distribution of depressive symptoms. Third, since this study is a cross-sectional study, longitudinal studies are necessary to delineate the stability of the distribution of depressive symptoms during middle adulthood. Finally, although the curve for middle adulthood exhibits a linear pattern with a log-normal scale, we did not perform authentic curve fitting based on a mathematical model.

Despite these limitations, the present study provides important information regarding the nature of the distribution of depressive symptoms. The degree to which these results can be generalized to other populations or other scales for depressive symptoms is unclear but warrants examination.
